# Key Findings and Comparisons From Analogous Case-Cluster Studies for Dengue Virus Infection Conducted in Machala, Ecuador, and Kamphaeng Phet, Thailand

**DOI:** 10.3389/fpubh.2020.00002

**Published:** 2020-02-12

**Authors:** Kathryn B. Anderson, Anna M. Stewart-Ibarra, Darunee Buddhari, Efrain Felix Beltran Ayala, Rachel J. Sippy, Sopon Iamsirithaworn, Sadie J. Ryan, Stefan Fernandez, Richard G. Jarman, Stephen J. Thomas, Timothy P. Endy

**Affiliations:** ^1^Department of Medicine, SUNY Upstate Medical University, Syracuse, NY, United States; ^2^Armed Forces Research Institute of Medical Science, Bangkok, Thailand; ^3^Department of Microbiology and Immunology, SUNY Upstate Medical University, Syracuse, NY, United States; ^4^Institute for Global Health and Translational Science, SUNY Upstate Medical University, Syracuse, NY, United States; ^5^Department of Montevideo, Inter-American Institute for Global Change Research (IAI), Montevideo, Uruguay; ^6^Carrera de Medicina de la Universidad Técnica de Machala, Machala, Ecuador; ^7^Department of Geography, University of Florida, Gainesville, FL, United States; ^8^Ministry of Public Health (Thailand), Nonthaburi, Thailand; ^9^Emerging Pathogens Institute, University of Florida, Gainesville, FL, United States; ^10^Walter Reed Army Institute of Research, Silver Spring, MD, United States

**Keywords:** dengue, epidemiology, observational study, Thailand, Ecuador

## Abstract

Dengue viruses (DENV) pose a significant and increasing threat to human health across broad regions of the globe. Currently, prevention, control, and treatment strategies are limited. Promising interventions are on the horizon, including multiple vaccine candidates under development and a renewed and innovative focus on controlling the vector, *Aedes aegypti*. However, significant gaps persist in our understanding of the similarities and differences in DENV epidemiology across regions of potential implementation and evaluation. In this manuscript, we highlight and compare findings from two analogous cluster-based studies for DENV transmission and pathogenesis conducted in Thailand and Ecuador to identify key features and questions for further pursuit. Despite a remarkably similar incidence of DENV infection among enrolled neighborhood contacts at the two sites, we note a higher occurrence of secondary infection and severe illness in Thailand compared to Ecuador. A higher force of infection in Thailand, defined as the incidence of infection among susceptible individuals, is suggested by the higher number of captured *Aedes* mosquitoes per household, the increasing proportion of asymptomatic infections with advancing age, and the high proportion of infections identified as secondary-type infections by serology. These observations should be confirmed in long-term, parallel prospective cohort studies conducted across regions, which would advantageously permit characterization of baseline immune status (susceptibility) and contemporaneous assessment of risks and risk factors for dengue illness.

## Introduction

Infection with dengue viruses (DENV) is responsible for a significant burden of disease across tropical and subtropical regions of the globe. However, the history and epidemiology of DENV clearly differ between Asia and the Americas. Asia has been hyperendemic for all four DENV serotypes for decades and consistently demonstrates one of the highest burdens of dengue-related disease in the world (1). In contrast, in the Americas, following the abandonment of successful mosquito control programs in the 1960s, DENV serotypes were sequentially reintroduced into circulation. In Ecuador, DENV re-emerged in 1988, co-circulation of all four DENV serotypes was documented in 2000, and the first cases of severe dengue were seen in 2001 (2, 3).

The nature and extent of differences in DENV transmission intensity and clinical manifestations of disease between Asia and the Americas remain poorly understood. Observational cohort studies conducted in multiple regions of the globe have contributed significantly to our understanding of DENV transmission and pathogenesis (4–7), however, differences in study-specific aims and methodologies have largely precluded direct comparisons across regions. Limited regionally-comparative analyses of DENV seroprevalence (8) and mean ages of infection (9) suggest that the force of infection (incidence among susceptible individuals) may be higher, on average, in Asia than in the Americas. Relatedly, presumed higher levels of susceptibility to DENV serotypes in the Americas compared to Asia may indicate the potential for DENV epidemics for more explosive epidemics of greater magnitude in the Americas.

There is currently no licensed antiviral for DENV infection and the only DENV vaccine currently licensed for use in multiple countries has recently generated safety concerns due to an increased risk of hospitalized illness observed in DENV-naïve vaccine recipients (10). Currently-available vector control measures have been ineffective in stopping the transmission of Aedes-transmitted pathogens (including DENV) (11), however, pioneering methods of innovative vector control such as the field release of Wolbachia-infected *Aedes* mosquitoes (12) and transgenic mosquitoes (13) offer promise. The effective evaluation and implementation of novel DENV vaccines and vector control measures would benefit from an improved understanding of the similarities and differences in DENV transmission and pathogenesis across continents.

Multiple recent epidemics of DENV and other arboviruses spread by *Aedes* vectors across diverse regions of the globe underscore the urgent need to better understand the patterns and drivers of arboviral disease transmission in order to prepare for the epidemics to come. In this manuscript, we summarize and contrast findings from two analogous cluster investigation studies conducted in Machala, Ecuador, and Kamphaeng Phet, Thailand, to highlight possible distinguishing features in DENV epidemiology between the two regions and to identify important avenues for future research.

## Materials and Methods

### Summary

**Figure 1:**
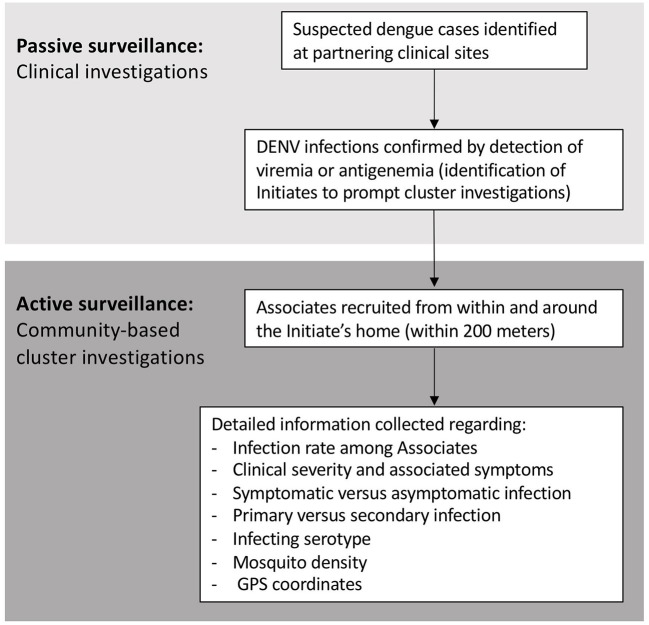
Summary of study activities for the Thai and Ecuadorian studies.

Analogous case-cluster surveillance studies were conducted in Kamphaeng Phet (KPP), Thailand, from 2009 to 2012, and Machala, Ecuador, from 2014 to 2015. Both studies utilized enhanced passive surveillance to identify and recruit patients with suspected dengue illnesses presenting to local participating clinics and hospitals (Figure 1). Subsequent confirmation of a DENV infection prompted the further study of individuals residing within and around the home of the infected individual. Active surveillance was then used to identify symptomatic and asymptomatic infections occurring within enrolled neighborhood contacts. The detailed methods for both studies have been published previously (14, 15). Detailed comparisons of the surveillance and diagnostic methods are presented in the text below and Table 1.

**Table 1:** Study-specific methods in KPP, Thailand, and Machala, Ecuador.

	**KPP, Thailand**	**Machala, Ecuador**
**Identification of initiates**		
Locations/s of recruitment	Kamphaeng Phet Provincial Hospital (inpatients)	Inpatients and outpatients presenting to MOH clinics and hospitals
Inclusion criteria	• Age >6 months • DENV infection confirmed by RT-PCR	• Age >6 months • Clinical diagnosis of suspected dengue
**Identification of associates**		
Eligible homes	All homes located within 200m of Initiate's home, with reported fever in preceding 7 days	5 homes: the Initiate's home and one each located in the four cardinal directions (N, S, E, W)
Inclusion criteria	• Age >6 months • Residing in a house with a history of reported fever in the prior week	• Age >6 months • Residing in the Initiate's home or in the four houses located N, S, E, W
Follow-up	Specimens and data collected on days 0 and 15	Specimens and data collected on day 0 only
**Laboratory diagnostic methods**		
Molecular	• DENV RT-PCR (16)	• DENV NS1 rapid test (PanBio) • Qualitative DENV rtRT-PCR (17) • CHIKV and ZIKV RT-PCR
Serological	• DENV and JEV IgM and IgG ELISA (paired specimens) (18)	• DENV IgM and IgG ELISA (PanBio)

### Study Sites

Kamphaeng Phet province is located in northern Thailand, with a moderately dense urban center (Muang) surrounded by agricultural zones. Machala is the capital of El Oro province, located in southern coastal Ecuador; the town is densely populated and surrounded by agriculture and aquaculture areas. The study sites are comparable in total population, elevation, gross domestic product (GDP), and the co-circulation of all four DENV types (Table 2). Both regions experience the co-circulation of multiple other arboviruses in addition to DENV. Chikungunya virus (CHIKV) emerged in Ecuador at the end of 2014, and the first confirmed instances of autochthonous Zika virus (ZIKV) transmission in Ecuador were reported in January 2016 (15). Neither ZIKV nor CHIKV were known to be in circulation in northern Thailand at the time of the study, however there is increasing evidence for long-standing endemicity of ZIKV in the region (19). Both regions practice routine immunization of pediatric populations for non-DENV flaviviruses: Japanese encephalitis vaccine (JEV) is part of the Expanded Program on Immunization (EPI) in Thailand, with rates of coverage estimated to be 92% or higher (20), and yellow fever vaccine (YFV) vaccine is part of the EPI in Ecuador, with rates of coverage approaching 80% in certain high-risk areas (21).

**Table 2:** Comparison of key features of the field sites in Muang Kamphaeng Phet (KPP), Thailand, and Machala, Ecuador.

**Variables**	**KPP, Thailand**	**Machala, Ecuador**
Population	213,228	280,000
Location (lat, long)	Southeast Asia (16°28′ N, 99°31′ E)	Pacific coast of South America (3°15′ S, 79°57′ W)
Elevation	80 m	9 m
Land use	Moderately dense urban area surrounded by agricultural areas (rice)	Dense urban area surrounded by coastal mangroves, farming (bananas) and aquaculture (shrimp)
Climate	Tropical climate with marked rainy season: May to Oct (dengue season); avg max temp 33.5°C; avg min temp: 22.9°C	Tropical climate with marked rainy season: Feb to May (dengue season); avg max temp 29.1°C; avg min temp 22.1°C
Annual per capita GDP (2017 USD)	$6,594	$6,199
Dengue transmission	Endemic seasonal transmission, interannual outbreaks	
Arbovirus context	DENV is a top public health concern; ZIKV likely with long standing endemicity; JEV vaccination widespread	DENV is a top public health concern; CHIKV/ZIKV are new; YFV vaccination widespread
Dengue vectors	*A. aegypti and A. albopictus*	*A. aegypti*

### Definitions

Initiates are individuals who presented to participating clinical sites with suspected dengue illnesses, who were subsequently confirmed to have DENV infection. A subset of Initiates were randomly selected for participation in community-based cluster investigations. Associates are individuals residing within the Initiate's household or within a 200-m radius of the Initiate's household, who met study-specific enrollment criteria. Together, the Associate homes plus the Initiate's home made up a cluster.

### Recruitment and Surveillance of Initiates

- Thailand. Initiates were recruited from among individuals admitted to the public referral hospital, Kamphaeng Phet Provincial Hospital (KPPPH) with suspected dengue infection. Inclusion criteria were: age >6 months and blood drawn and RT-PCR performed to confirm DENV infection within 24 h of hospital admission. Acute and convalescent blood specimens were collected on enrollment and 15 (±5 days) thereafter.
- Ecuador. Initiates were recruited from among inpatients and outpatients presenting to four clinics operated by the Ministry of Health and the associated public referral hospital, Teófilo Dávila Hospital. Inclusion criteria were: age ≥6 months and a clinical diagnosis of suspected dengue. Acute blood specimens were collected on enrollment. A maximum of 4 Initiates were randomly selected each week to initiate cluster investigations.

### Recruitment and Surveillance of Associates

- Thailand. All individuals aged >6 months residing within the Initiate's household were invited to enroll. Further, all homes located within a 200-m radius of the Initiate's household were visited by the study team. If anyone in a given household reported fever within the previous 7 days, all residents of that household aged >6 months were invited to participate in the study, to a maximum to 25 Associates enrolled per cluster. Blood specimens were collected on the day of enrollment (“day 0”) and roughly 15 ± 5 days thereafter. If an Associate developed fever during the 15-day follow-up period, a second acute blood specimen was drawn and the period of follow-up shifted by an additional 15 days for that individual. Adult *Aedes* mosquitoes were collected from all homes within 200 m of the Initiate home using backpack aspirators.
- Ecuador. All individuals aged ≥6 months residing within the Initiate's household were invited to enroll. Further, all individuals aged ≥6 months and residing in households located in the cardinal directions from the Initiate household at a maximum distance of 200-m were invited to enroll. Thus, there was an imposed limit of five households per cluster (the Initiate's home, plus one home each located to the north, south, east, and west). Blood specimens were collected on the day of enrollment only (“day 0”). Adult *Aedes* mosquitoes were collected from the five enrolled homes (the Initiate's home and the four neighboring homes) using backpack aspirators.
- Geospatial data collection. For both sites, the locations (latitude, longitude) of all Initiate homes and all homes within 200-m of the Initiate home were recorded using handheld GPS devices.

### Laboratory Diagnostics

- Thailand. DENV RT-PCR and DENV and JEV IgM/IgG ELISA were used to identify DENV infections occurring in Initiates and Associates. The nested RT-PCR method described by Lanciotti et al. was used to detect DENV RNA and to identify the infecting serotype (16). AFRIMS in-house IgM and IgG ELISAs were used to serologically diagnose DENV infections and to discern DENV and JEV as described previously (18). RT-PCR and IgM/IgG ELISA were performed on the day 0 and 15 specimens for all enrolled Associates.
- Ecuador. DENV NS1 rapid strip tests (PanBio Dengue Early Rapid Test) were used to identify confirmed DENV infections (Initiates) from among ill patients at clinical sites. Qualitative real-time RT-PCR assays for DENV1-4 were performed as per the CDC DENV1-4 Real Time RT-PCR Assay (CDC, Catalog number KK0128) (17). Commercial ELISA kits (PanBio) were used to detect DENV IgM (Dengue Capture IgM) and IgG (Dengue Capture IgG). All Initiates and Associates from Ecuador also underwent testing for ZIKV and CHIKV by RT-PCR; these results have been previously presented and are not discussed here (15).

### Classification of DENV Infections

- Thailand. For the purposes of this analysis an acute or recent DENV infection in an Associate was defined as: (1) detection of DENV RNA in a specimen collected at any time point (e.g., from the day 0 and 15 visits as well as acute specimens collected in the setting of incident fever), or (2) detection of DENV IgM in any specimen, or (3) DENV IgM not detected but DENV IgG >100 and rising (acute infection) or decreasing (recent infection) in paired specimens. A primary infection was defined as an IgM/IgG ratio ≥1.8, a secondary infection as a ratio <1.8. A symptomatic DENV infection was defined as (1) an acute laboratory-confirmed DENV infection plus (2) the presence of one or more classical dengue symptom/s (e.g., fever, headache, muscle/joint pain, retro-orbital pain, abdominal pain, drowsiness/lethargy, rash). Clinical data were collected for Initiates and Associates during the day 0 and 15 visits, as well as during unscheduled visits prompted by reported fever, inquiring about any current and recent symptoms since the last study visit. An asymptomatic DENV infection was defined as (1) an acute laboratory-confirmed DENV infection plus (2) the absence of all of these symptoms during the entire period of follow-up (typically 15 ± 5 days).
- Ecuador. An acute DENV infection in an Associate was defined as the detection of DENV RNA by RT-PCR in the enrollment specimen (only a single specimen collected). A recent infection was defined as the detection of IgM in the enrollment specimen (and RT-PCR negative). A primary infection was defined as an IgM/IgG ratio ≥1.8, a secondary infection as a ratio <1.8. A symptomatic DENV infection was defined as (1) an acute laboratory-confirmed DENV infection plus (2) the presence of one or more classical dengue symptom/s (e.g., fever, headache, muscle/joint pain, retro-orbital pain, abdominal pain, drowsiness/lethargy, rash). Clinical data were collected for Associates at the time of enrollment only and reflected symptoms present at the time of enrollment or at any point during the preceding 7 days. An asymptomatic DENV infection was defined as (1) an acute laboratory-confirmed DENV infection plus (2) the absence of all of these symptoms at the time of interview and during the preceding 7 days.

### Ethics Statement

For the Ecuador study, the protocol was reviewed and approval by Institutional Review Boards (IRBs) at SUNY Upstate Medical University, the Human Research Protection Office (HRPO) of the U.S. Department of Defense, the Luis Vernaza Hospital in Guayaquil, Ecuador, and the Ecuadorean Ministry of Health. For the Thai study, the protocol was approved by the IRBs of the Thai Ministry of Public Health (MOPH), Walter Reed Army Institute of Research (WRAIR, protocol number 1526), and SUNY Upstate Medical University. The IRBs of the University of California, Davis (UCD), University of Rhode Island (URI), and University at Buffalo established relying agreements with WRAIR IRB. Prior to the start of the study, all participants engaged in a written informed consent or assent process as previously described (14, 15).

## Results

### Characteristics of Enrolled Initiates and Associates

Three hundred twenty-three Initiates were enrolled in Thailand between November 2009 and November 2012 (Table 3), with enrollment thus capturing three peak periods for DENV transmission (i.e., the rainy season), which variably reaches its maximum in July-August and wanes in October–November each year. Forty-four Initiates were enrolled in Ecuador between January 2014 and June 2015, with enrollment thus spanning two peak periods for DENV transmission, which typically reaches its maximum in March-May and wanes in June–July each year (22). All four DENV serotypes were detected in Initiates in Thailand, during the study period, while only DENV-1 and DENV-2 were detected among Initiates in Ecuador. 26.4% of Initiates in Ecuador were RT-PCR negative, with DENV infection confirmed by NS1 rapid test or NS1 ELISA. The median ages of Initiates in Ecuador and Thailand were similar (16 and 14.5 years, respectively). Only 1.9% of Initiates in Thailand had primary DENV infections by serology vs. 25.0% in Ecuador. By definition, 100% of Initiates were derived from hospitalized illnesses in Thailand, while 25.0% were hospitalized in Ecuador.

**Table 3:** Features of Initiates in Thailand and Ecuador.

	**KPP**	**Machala**
Number of initiates	323	44
Median age in years (range)	16 (2–72)	14.5 (1–67)
% female	48.6%	38.6%
DENV serotype		
DENV-1	23.2%	25.0%
DENV-2	60.4%	40.9%
DENV-3	11.5%	0%
DENV-4	5.0%	0%
Not detected^*^	0%	26.4%
% primary	1.9%	25.0%^**^
% hospitalized	100.0%	25.0%

* *NS1 rapid tests were used to identify Initiate cases in Ecuador; thus, not all were RT-PCR positive*.

** *Among those with valid serology (68.1% or 30/44)*.

One thousand two hundred forty-two Associates were enrolled in Thailand and 384 in Ecuador, with a median of 3 and 8 Associates per Initiate in each country, respectively (Table 4). Overall, relatively few Associates reported a history of JEV (in Thailand) or YFV vaccination (in Ecuador); however, 96.1% of Thai children (aged <18 years) reported a history of JEV vaccination and 19.6% of Ecuadorian children reported a history of YFV vaccination. Three hundred three Associates in Thailand (24.3%) and 96 in Ecuador (25.0%) were confirmed to have acute or recent DENV infection. Eliminating the results from the convalescent blood draw from the Thailand data (e.g., forcing a mirroring of study methods for the two sites by considering only the diagnostics testing results from the enrollment specimen), the number of infected Associates detected in the Thai study decreased to 202 (data not shown). Thus, extending the surveillance period by 15 days and incorporating a convalescent blood draw increased the detection of DENV infections in Associates by 33% (from 202 to 303 infections).

**Table 4:** Features of enrolled Associates in Thailand and Ecuador.

	**KPP**	**Machala**
Number	1,246	384
Median number of Associates per Initiate	3 (1–17)	8 (4–17)
Median age in years (range)	30 (0–96)	34 (0–87)
% female	57.4%	65.9%
History of flavivirus vaccine^*^	18.5%	11.7%
Total % with acute or recent infection	24.3%	25.0%
Within Initiate's house	22.0%	29.1%
Neighboring house^**^	26.8%	23.9%
RT-PCR positive (*n*)	44.2%	43.2%
Among RT-PCR positive Associates, infecting DENV serotype		
DENV-1	24.6%	15.8%
DENV-2	50.0%	57.9%
DENV-3	20.9%	26.3%
DENV-4	4.5%	0%
% concordant with Initiate serotype	94.0%	66.7%
% primary	11.8%	39.6%
% asymptomatic	25.1%	33.3%
Mean # adult *Aedes* females/house	1.67 (3.41)	0.95 (1.62)
Homes with infections	1.90 (3.83)	1.00 (1.76)
Homes without infections	0.99 (1.75)	0.80 (1.16)

* *Reported history of JEV vaccine (Thailand) or YFV vaccine (Ecuador)*.

** *Note that for Thailand, Associate houses were enrolled out to a radius of 200 m if anyone in the home reported a history of recent fever; for Ecuador, Associate houses were enrolled in the four cardinal directions and 94% were within 100 m of the Initiate house (15)*.

**Figure 2:**
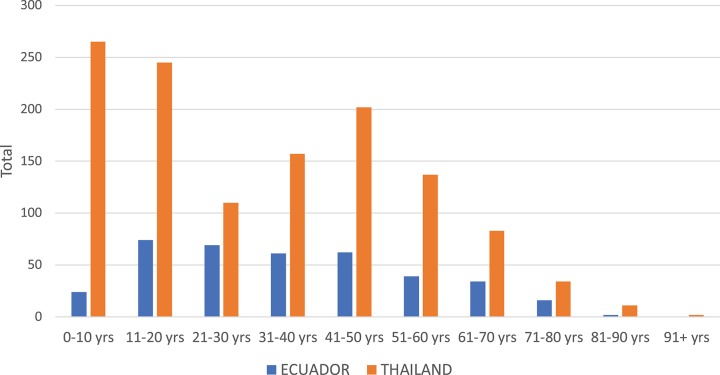
Histogram of ages of enrolled Associates in Thailand (orange) and Ecuador (blue).

In Thailand, the DENV serotype detected in Associates matched the Initiate's serotype 94.0% of the time. In contrast, concordance was only 66.7% in Ecuador (i.e., one in three Associates were infected with a different serotype than the Initiate for that cluster). The clear majority of DENV infections in Associates in Thailand and Ecuador were symptomatic (defined as the report of any symptom within 7 days of enrollment through the 15-day follow-up for Thailand and within the past 7 days from enrollment for Ecuador). The mean number of *Aedes* females per home was higher in Thailand than in Ecuador (*p* = 0.034 by Mann Whitney *U*-test); for both sites, albeit non-significantly, the number of mosquitoes captured was higher in Associate homes with identified DENV infections than in those without DENV infections (*p* > 0.05 for both comparisons). In Thailand, the largest number of Associates was enrolled in the age group comprising children aged 0–10 years (Figure 2). In Ecuador, the largest age group enrolled comprised individuals aged 11–20 years.

### Characteristics of DENV Infections in Associates

**Figure 3:**
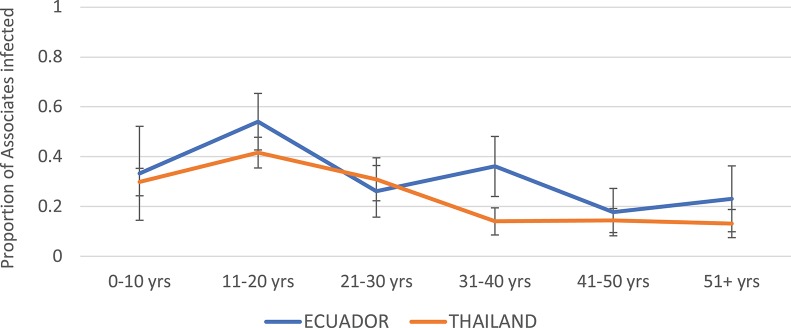
Proportion of Associates confirmed to have DENV infection, by age and study site. Thailand is shown in orange and Ecuador in blue. Error bars reflect the 95% confidence intervals for the proportions.

The highest infection rates among Associates for both Thailand and Ecuador were observed in the age group 11–20 years (Figure 3). The rates were roughly similar but generally higher for Ecuador than for Thailand. Incidence rate decreased with age at both sites.

**Figure 4:**
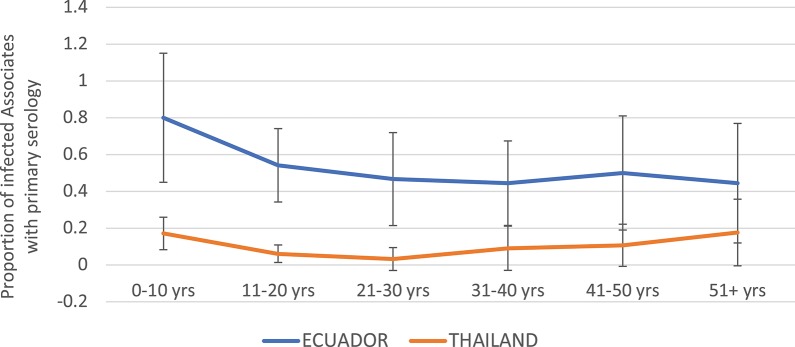
Proportion of DENV-infected Associates found to have primary DENV infection (by ELISA), by age and study site. Thailand is shown in orange and Ecuador in blue. Error bars reflect the 95% confidence intervals for the proportions.

The proportion of DENV infections that were primary by serology (EIA) was much higher in Ecuador than in Thailand overall (Figure 4). In the age group 0–10 years, 80.0% of DENV infections were primary in Ecuador, vs. 17.1% in Thailand. In Ecuador, the proportion of infections that were primary generally decreased with age, while in Thailand the proportion remained relatively level between 0 and 20%.

**Figure 5:**
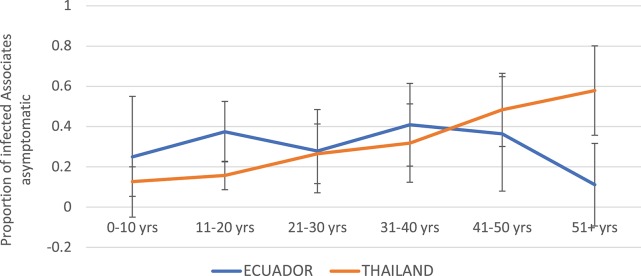
Proportion of DENV-infected Associates with asymptomatic infection (i.e., denying any of the solicited symptoms) by age and study site. Thailand is shown in orange and Ecuador in blue. Error bars reflect the 95% confidence intervals for the proportions.

Children were more likely to experience symptomatic infection in Thailand as compared to Ecuador (Figure 5). The proportion of DENV infections that were asymptomatic increased steadily with age in Thailand, while in Ecuador the proportion asymptomatic remained relatively level between 15 and 40%.

### Symptoms of DENV Infection in Associates

In Thailand, children were more likely than adults to report fever, headache, upper respiratory symptoms (rhinorrhea, cough), and abdominal symptoms (pain, nausea/vomiting) (Table 5). Children were also more likely to be hospitalized. Individuals experiencing a secondary DENV infection were more likely to be hospitalized and to demonstrate all symptoms solicited (significant for headache, anorexia, nausea/vomiting, drowsiness, muscle/joint pain, and abdominal pain). Children experiencing secondary infection reported the highest frequency of symptoms, notably with 82.9% reporting fever and 25.6% becoming hospitalized.

**Table 5:** Symptoms reported by enrolled Associates by age (adult = age ≥ 18 years, child = age ≤ 18 years), among those with symptomatic infections (defined as the presence of any solicited symptom) in Thailand.

	**Thailand**											
	**Total—by age**			Total—by serology^*^			**Primary**			**Secondary**		
	**Child**	**Adult**	***p*-value**	**1^**o**^**	**2^**o**^**	***p*-value**	**Child**	**Adult**	***p*-value**	**Child**	**Adult**	***p*-value**
# Dengue illnesses^**^	163	140	NA	32	238	NA	17	15	NA	129	109	NA
Asymptomatic	13.5%	38.6%	<0.001	37.5%	21.4%	0.044	17.6%	60.0%	0.027	10.9%	33.9%	<0.001
Hospitalized	21.5%	7.9%	<0.001	3.1%	18.5%	0.024	5.9%	0.0%	1.000	25.6%	10.1%	0.002
Fever	80.4%	51.4%	<0.001	59.4%	69.3%	0.312	76.5%	40.0%	0.070	82.9%	53.2%	<0.001
Headache	56.4%	42.1%	0.016	15.6%	56.7%	<0.001	11.8%	20.0%	0.645	65.9%	45.9%	0.002
Rhinorrhea	30.1%	6.4%	<0.001	15.6%	20.6%	0.641	23.5%	6.7%	0.338	31.8%	7.3%	<0.001
Cough	35.6%	15.0%	<0.001	15.6%	28.6%	0.141	11.8%	20.0%	0.645	39.5%	15.6%	<0.001
Anorexia	46.6%	26.4%	<0.001	18.8%	39.9%	0.020	23.5%	13.3%	0.659	51.2%	26.6%	<0.001
Nausea/vomiting	42.3%	19.3%	<0.001	6.3%	33.6%	0.001	11.8%	0.0%	0.486	45.0%	20.2%	<0.001
Drowsiness	30.7%	21.4%	0.089	6.3%	29.4%	0.005	53.1%	46.9%	0.212	34.9%	22.9%	0.047
Muscle/joint pain	38.7%	45.7%	0.243	15.6%	47.5%	0.001	5.9%	26.7%	0.161	47.3%	47.7%	1.000
Abdominal pain	27.0%	11.4%	0.001	6.3%	23.1%	0.035	0.0%	13.3%	0.212	32.6%	11.9%	<0.001
Rash	12.3%	7.9%	0.255	9.4%	11.3%	1.000	17.6%	0.0%	0.229	13.2%	9.2%	0.413
Diarrhea	19.6%	11.4%	0.059	9.4%	16.4%	0.437	11.8%	6.6%	1.000	20.2%	11.9%	0.113
Retroorbital pain	17.8%	24.3%	0.201	9.4%	21.8%	0.158	5.9%	13.3%	0.589	20.9%	22.9%	0.754
Bleeding	6.7%	2.9%	0.183	0.0%	5.9%	0.386	0.0%	0.0%	NA	8.5%	2.8%	0.094

* *Of those with serologically-confirmed DENV infection (i.e., either primary or secondary DENV infection)*.

** *Among all those with confirmed DENV infection (i.e., whether symptomatic or asymptomatic)*.

*P-values < 0.05 indicate statistically significant comparisons, applying Mantel-Haenszel chi-squared testing*.

*NA indicates not applicable*.

In Ecuador, children experiencing DENV infection were more likely than adults to report rash, and adults were more likely than children to report muscle and joint pain (Table 6). Symptoms were more common in Associates experiencing secondary DENV infections. In Ecuador, most symptoms were more common in Associates experiencing secondary DENV infections although there was limited power to detect significant associations given low numbers. Rash was more common in children experiencing primary DENV infection.

**Table 6:** Symptoms reported by enrolled Associates by age (adult = age ≥ 18 years, child = age ≤ 18 years), among those with symptomatic infections (defined as the presence of any solicited symptom) in Ecuador.

	**Ecuador**											
	**Total—by age**			Total—by serology^*^			**Primary**			**Secondary**		
	**Child**	**Adult**	***p*-value**	**1^**o**^**	**2^**o**^**	***p*-value**	**Child**	**Adult**	***p*-value**	**Child**	**Adult**	***p*-value**
# Dengue illnesses^**^	27	67	NA	42	45	NA	13	29		8	37	
Asymptomatic	34.2%	32.9%	0.889	42.9%	22.2%	0.040	53.8%	37.9%	0.501	25.0%	21.6%	1.000
Hospitalized	0%	0%	NA				0%	0%	NA	0%	0%	NA
Fever	22.9%	14.1%	0.282	9.8%	20.0%	0.235	8.3%	10.3%	1.000	25.0%	18.9%	0.651
Headache	33.3%	27.8%	0.660	19.5%	37.8%	0.095	16.7%	20.7%	1.000	25.0%	40.5%	0.690
Rhinorrhea	N/A	N/A	N/A	N/A	N/A	N/A	N/A	N/A	N/A	N/A	N/A	N/A
Cough	N/A	N/A	N/A	N/A	N/A	N/A	N/A	N/A	N/A	N/A	N/A	N/A
Nausea/vomiting	13.2%	7.6%	0.333	4.8%	8.9%	0.677	0.0%	6.9%	1.000	0.0%	10.8%	1.000
Drowsiness	16.7%	21.5%	0.623	14.6%	26.7%	0.195	8.3%	17.2%	0.651	12.5%	29.7%	0.419
Muscle/joint pain	11.1%	34.2%	0.012	31.7%	33.3%	1.000	25.0%	34.5%	0.719	12.5%	37.8%	0.236
Abdominal pain	13.9%	22.8%	0.323	17.1%	26.7%	0.311	8.3%	20.7%	0.651	25.0%	27.0%	1.000
Rash	22.2%	7.6%	0.034	14.6%	13.3%	1.000	33.3%	6.9%	0.050	25.0%	10.8%	0.286
Diarrhea	2.8%	11.4%	0.168	4.9%	15.6%	0.161	0.0%	6.9%	1.000	0.0%	18.9%	0.321
Retroorbital pain	13.9%	26.6%	0.155	17.1%	35.6%	0.087	16.7%	17.2%	1.000	25.0%	37.8%	0.691
Bleeding	0.0%	1.3%	1.000	2.4%	0.0%	0.477	0.0%	3.4%	1.000	0.0%	0.0%	NA

* *Of those with valid serological results (i.e., either primary or secondary DENV infection)*.

** *Among all those with confirmed DENV infection (i.e., whether symptomatic or asymptomatic)*.

*P-values < 0.05 indicate statistically significant comparisons, applying Mantel-Haenszel chi-squared testing*.

*NA indicates not applicable*.

## Discussion

DENV pose a significant and increasing threat to human health across broad regions of the globe. Counter measures to prevent human exposure to infected *Aedes* mosquitoes and to prevent illness, once exposed, are urgently needed. With promising interventions on the horizon, including multiple vaccine candidates under development (23) and a renewed and innovative focus on the vector, *Aedes aegypti* (12, 13), there persist significant gaps in our understanding of the similarities and differences in DENV epidemiology across regions of potential implementation and evaluation. In this manuscript, we highlight and compare findings from two analogous cluster-based studies for DENV transmission and pathogenesis conducted in Thailand and Ecuador to identify key features and questions for further pursuit.

**Figure 6:**
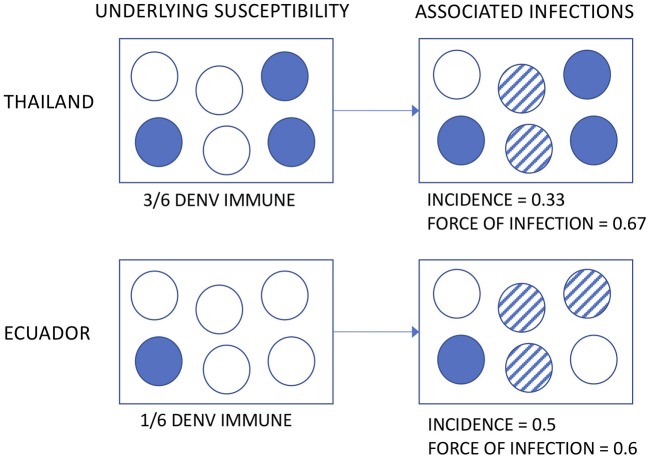
Hypothetical illustration of the possible relationships between underlying susceptibility to DENV, observed incidence, and force of infection.

The incidence of DENV infection among Associates was remarkably similar across age groups in both countries. Applying the same definition to the Thai Associates as to the Ecuadorian Associates (i.e., based upon the enrollment specimen only), the incidence rate in Ecuador was at least 33% higher. This is somewhat surprising, given prior estimates suggesting a higher transmission intensity in Asia than in the Americas (8, 9). Multiple possible explanations exist for this. First, the incidence of DENV has been shown to vary significantly in time and space (24). The studies were conducted at different time points and for relatively short intervals and thus infection rates by country may be confounded by year. Further, the clinical severity and transmissibility of DENV has been demonstrated to vary by serotype (25–27). Second, the underlying susceptibility of the Associate populations is not known, by nature of the study design (i.e., cluster investigations based upon the identification of a DENV infection in a neighbor). If the Thai Associates had a higher level of pre-existing DENV immunity, a similar or even lower incidence may still reflect a high force of infection (see hypothetical illustration in Figure 6). This distinction is important, because the force of infection directly translates to the risk experienced by DENV-naïve subjects visiting or born into an area as well as the level of coverage needed by interventions to decrease transmission.

Interestingly, intra-cluster concordance of DENV serotypes between Initiates and Associates was only 67% in Ecuador, as compared to 94% in Thailand. This focal, concurrent micro-circulation of multiple serotypes has not, to our knowledge, been documented previously. Potential explanations for this finding may include: greater population-level immunological bottle necks for serotype co-circulation in Thailand, resulting from many decades of hyperendemic transmission, differences in human movement patterns, and/or differences in the spatial scale or hot-spots for transmission between the two sites. Long-term, parallel prospective cohort studies conducted across regions would contribute significantly to our understanding of DENV epidemiology, permitting characterization of baseline immune status (susceptibility), shifts in DENV serotypes and genotypes over time, and diverse risk factors for DENV infection and dengue illness.

The incidence of symptomatic DENV infection among Associates for both studies was much higher than has been reported in previous prospective cohort studies, at 25 and 33% for Thailand and Ecuador, respectively (7, 28) This may reflect recall bias, wherein individuals enrolled in cluster studies are more likely to notice and report even minor symptoms given that a neighbor has recently been diagnosed with a DENV infection. It is also possible that the enrollment of ill Initiates at their point of entry into the healthcare system imposes a sampling bias, selecting for more severe DENV serotypes, genotypes, and/or strains with an increased ability to infect and cause disease in Associates. Finally, it should be noted that the case definition for “symptomatic infection” applied in this analysis is more sensitive than the definition applied in some other studies. For example, prior analyses from KPP have required the presence of fever to define symptomatic illness, a symptom reported by 89% of DENV-infected Thai Associates with any clinical symptoms and only 24% of symptomatic, DENV-infected Ecuadorian Associates in the current analyses. This suggests that using fever as the sole criterion for “symptomatic infection” in field studies for DENV may result in the misclassification of potentially large numbers of ill subjects as “asymptomatic.” Interestingly, the incidence of asymptomatic DENV infection increased with age in Thailand but remained relatively flat in Ecuador; this may reflect a higher force of infection for DENV in Thailand, with accumulated cross-protective immunity through multiple DENV exposures over time.

The clinical severity and manifestations of DENV infection in Associates differed between Thailand and Ecuador. 21.5% of children and 7.9% of adults enrolled as Associates in Thailand were hospitalized with dengue illnesses, as compared to 0% in Ecuador. This may reflect the greater occurrence of secondary DENV infection in Thai children, for whom rates of hospitalization were 25.6% and for whom most clinical symptoms were also more common (fever, headache, abdominal symptoms, etc.). Other possibilities for the greater clinical severity in Thailand include differences in the virulence of circulating DENV between regions and/or differences in study design, given that Thai Associate households were enrolled on the basis of reported fever and Ecuadorian households simply on the basis of their location relative to the Initiate house. Region-specific differences in patterns of care-seeking and criteria for hospitalization likely exist and may bias our comparisons; for example, individuals in Thailand may have been less likely to seek care for milder dengue illnesses as compared to individuals in Ecuador, and/or more likely to be hospitalized for a given clinical presentation. It is likely that human immunogenetic differences influence the clinical outcome to DENV infection and will differ across populations (29). Finally, undetected parasitic co-infections may play a role in modulating the immune response and thus the clinical outcome of DENV infection (30); for example, it is possible (but currently untested) that helminthic infections are more common in Ecuador than in Thailand and/or other parasitic co-infections such as *Trypanosoma cruzi* in the Americas may shape the clinical outcome of DENV infection.

The proportion of DENV infections identified as primary by DENV serology was much lower among similarly aged children in Thailand compared to Ecuador. This is presumably a reflection of the high rates of coverage for JEV vaccination in Thai children, manifesting as an anamnestic, secondary-type response to primary DENV infection. YFV and JEV are both well-known to cross-react with DENV in serological assays. Prior analyses from KPP suggest that prior JEV immunity may predispose toward symptomatic DENV infection (31); the potential for YFV to shape the clinical outcome of DENV infection is unknown. Potential differences in the force of infection for ZIKV between the Americas and Asia remain poorly understood, though there is increasing evidence that ZIKV has been endemic in Thailand, possibly at low levels, for decades (19). Serological cross-reactivity between DENV and ZIKV currently complicates the reliability of serologically-confirming infections due to either virus (32), however, assays promising improved specificity are in development and under validation (33, 34). Future studies should seek to further clarify the potential for exposure (natural or vaccine-derived) to a range of non-DENV flaviviruses to modulate the clinical and immunological outcomes of DENV infection; this knowledge will have particular relevance when evaluating the immunogenicity and efficacy of DENV vaccines across regions.

In addition to addressing the questions above, long-term, parallel prospective cohort studies would allow valuable characterization of larger patterns in DENV transmission across regions. The average age of DENV infection has been increasing in Thailand and other parts of Asia, indicating a decreased force of infection possibly due to a demographic transition toward an older population (35, 36). While Thailand has had all four DENV serotype in circulation for many decades, DENV is re-emerging in the Americas, and Ecuador became hyperendemic for all four serotypes as recently as 2000. It is, therefore, conceivable that Thailand may represent the future for Ecuador with regards to long-standing hyperendemic DENV transmission, and Ecuador, in turn, the future for areas that are currently DENV-naïve or low-endemicity but at risk for DENV introduction or expansion with climate change.

## Data Availability Statement

The datasets generated for this study are available on request to the corresponding author and with completion of appropriate regulatory requirements.

## Ethics Statement

The studies involving human participants were reviewed and approved by for the Ecuador study, the protocol was reviewed and approval by Institutional Review Boards (IRBs) at SUNY Upstate Medical University, the Human Research Protection Office (HRPO) of the U.S. Department of Defense, the Luis Vernaza Hospital in Guayaquil, Ecuador, and the Ecuadorean Ministry of Health. For the Thai study, the protocol was approved by the IRBs of the Thai Ministry of Public Health (MOPH), Walter Reed Army Institute of Research (WRAIR), and SUNY Upstate Medical University. The IRBs of the University of California, Davis (UCD), University of Rhode Island (URI), and University at Buffalo established relying agreements with WRAIR IRB. Written informed consent to participate in this study was provided by the participants' legal guardian/next of kin.

## Author Contributions

KA and AS-I performed the comparative analyses and drafted the manuscript. AS-I, EB, SR, and TE participated in the design, conduct, and analysis of the field study in Ecuador. DB, ST, RJ, and TE participated in the design and conduct of the field study in Thailand. TE was the PI for the NIH-funded R01 that provided support for the Thai study. SI provided support and guidance from the Thai Ministry of Public Health. SF provided support for the current analysis of the Thai data as current head of the department of virology, AFRIMS. All authors contributed to manuscript revision, read, and approved the submitted version.

### Conflict of Interest

The authors declare that the research was conducted in the absence of any commercial or financial relationships that could be construed as a potential conflict of interest.
